# Diseases of the Nose, Pharynx and Ear

**Published:** 1902-08

**Authors:** 


Diseases of the Nose, Pharynx and Ear. By Henry Gradle, M. D., Professor of Ophthalmology and Otology, Northwestern University Medical School, Chicago. Handsome octavo of 547 pages, profusely illustrated, including two full-page plates in colors. Philadelphia and London: W. B. Saunders & Co. 1902. Cloth, $3.50 net.

The author claims that this volume is intended to present diseases of the nose, pharynx and ear as he has seen them during an experience extending over twenty-five years of practice. Certain it is that the book is more than a mere compilation. It is essentially practical and is, therefore, worth having on one's shelf as a working guide. The author's clear, incisive style and happy choice of words makes it sure that no one will find the text dull or hard to understand.

The illustrations are only fair. In this particular many others of the new works seem to have an advantage. R.

